# Recent Advancements and Development in Nano-Enabled Agriculture for Improving Abiotic Stress Tolerance in Plants

**DOI:** 10.3389/fpls.2022.951752

**Published:** 2022-07-11

**Authors:** Natasha Manzoor, Liaqat Ali, Temoor Ahmed, Muhammad Noman, Muhammad Adrees, Muhammad Shafiq Shahid, Solabomi Olaitan Ogunyemi, Khlode S. A. Radwan, Gang Wang, Haitham E. M. Zaki

**Affiliations:** ^1^Department of Soil and Water Sciences, China Agricultural University, Beijing, China; ^2^University of Agriculture, Faisalabad, Vehari, Pakistan; ^3^Institute of Biotechnology, Zhejiang University, Hangzhou, China; ^4^Department of Environmental Sciences, Government College University Faisalabad, Faisalabad, Pakistan; ^5^Department of Plant Sciences, College of Agricultural and Marine Sciences, Sultan Qaboos University, Muscat, Oman; ^6^Department of Crop Protection, Federal University of Agriculture Abeokuta, Abeokuta, Nigeria; ^7^Plant Pathology Department, Faculty of Agriculture, Minia University, El-Minia, Egypt; ^8^National Black Soil and Agriculture Research, China Agricultural University, Beijing, China; ^9^Horticulture Department, Faculty of Agriculture, Minia University, El-Minia, Egypt; ^10^Applied Biotechnology Department, University of Technology and Applied Sciences-Sur, Sur, Oman

**Keywords:** abiotic stresses, drought, heavy metals, salinity, nanofertilizers

## Abstract

Abiotic stresses, such as heavy metals (HMs), drought, salinity and water logging, are the foremost limiting factors that adversely affect the plant growth and crop productivity worldwide. The plants respond to such stresses by activating a series of intricate mechanisms that subsequently alter the morpho-physiological and biochemical processes. Over the past few decades, abiotic stresses in plants have been managed through marker-assisted breeding, conventional breeding, and genetic engineering approaches. With technological advancement, efficient strategies are required to cope with the harmful effects of abiotic environmental constraints to develop sustainable agriculture systems of crop production. Recently, nanotechnology has emerged as an attractive area of study with potential applications in the agricultural science, including mitigating the impacts of climate change, increasing nutrient utilization efficiency and abiotic stress management. Nanoparticles (NPs), as nanofertilizers, have gained significant attention due to their high surface area to volume ratio, eco-friendly nature, low cost, unique physicochemical properties, and improved plant productivity. Several studies have revealed the potential role of NPs in abiotic stress management. This review aims to emphasize the role of NPs in managing abiotic stresses and growth promotion to develop a cost-effective and environment friendly strategy for the future agricultural sustainability.

## Introduction

The world’s population is expected to increase by 2 billion persons by 2050, inflating the food demands to feed the growing population (Mora et al., 2020; Zsögön et al., 2022). However, the global warming and climate change with an ever-increasing population, serve as the predominant limiting factors that hinder the efforts to meet the word’s food demand (Lowry et al., 2019). In the last few years, adverse climatic changes have induced abiotic stresses, such as heavy metal (HMs) toxicity, drought, heat, and high soil salinity, that lead to reduced crop productivity worldwide (Rizwan et al., 2012; Fahad et al., 2017). HMs in agricultural soils resulting from the large-scale application of chemical fertilizers, atmospheric deposition, sewage sludge, and rapid industrial growth. In recent decades, crop production has been continually effected by toxic HMs (Ahmed et al., 2021a; Chen et al., 2022). The high concentrations of HMs can reduce the plant growth by disrupting the nutrient uptake, antioxidant enzymes, photosynthetic machinery, and by increasing the reactive oxygen species (ROS) production (Adrees et al., 2021; Rizwan et al., 2021).

Soil salinity negatively affects at a physiological level, which disturbs ionic and water homeostasis. At the cellular level, salinity stress contributes to high accumulation of ROS that disturbs the cellular redox homeostasis (Abdel Latef et al., 2018). In addition, heat stress also causes ROS generation, which invariably impacts oxidative activities (Hasanuzzaman et al., 2012). Furthermore, prolonged drought has been reported to cause the reduction in stomatal opening, leaf size, leaf water potential, root growth, and seed number, size, and tolerance, inhibiting flowering, fruiting, and thus reduce crop production (Xu et al., 2016). Different approaches have been used to control abiotic stresses in plants, including conventional breeding, marker-assisted breeding, and transgenic crop engineering (Ashraf, 2010; Grover et al., 2013).

Among others, nano-enabled approaches have recently emerged as a promising tool to control nutrient deficiency, increase crop yields, transform biological systems, and management of plant stresses imposed by environment (Dimkpa et al., 2017; White and Gardea-Torresdey, 2018). Moreover, the use of nanoparticles (NPs) as nanofertilizers for targeted delivery of micronutrients is considered as efficient, cost-effective, eco-friendly, and best alternatives to chemical fertilizers (Irshad et al., 2021b; Shah et al., 2021). In the last few years, several methods, such as physical (electro-explosion, gamma radiations, pulse laser ablation, ion sputtering scattering, mechanical/ball milling), chemical (sol–gel, microemulsion, co-precipitation, hydrothermal) and biological approaches (using microorganisms/plants extracts), have been used for the production of metallic NPs (Ahmed et al., 2021d; Noman et al., 2022). Various studies have reported the application of NPs improved the abiotic stress tolerance in plants by modulating the biochemical, morpho-physiological and genetic mechanisms under salinity stress (Rossi et al., 2017), drought stress (Mozafari et al., 2018), and HMs stress (Bashir et al., 2020). Based on the revised literature, we conclude that the application of NPs against abiotic stress in crop production is considered as a reliable approach with long-term effectiveness compared with other traditional methods.

This review covers current opportunities for the potential application of NPs in agriculture and focuses on nano-enabled technologies for abiotic stress management. It further explores the possible NPs uptake mechanism and their positive effects on plant growth and soil fertility.

## Abiotic Stresses and Its Effect on Crop Production

**Figure 1:**
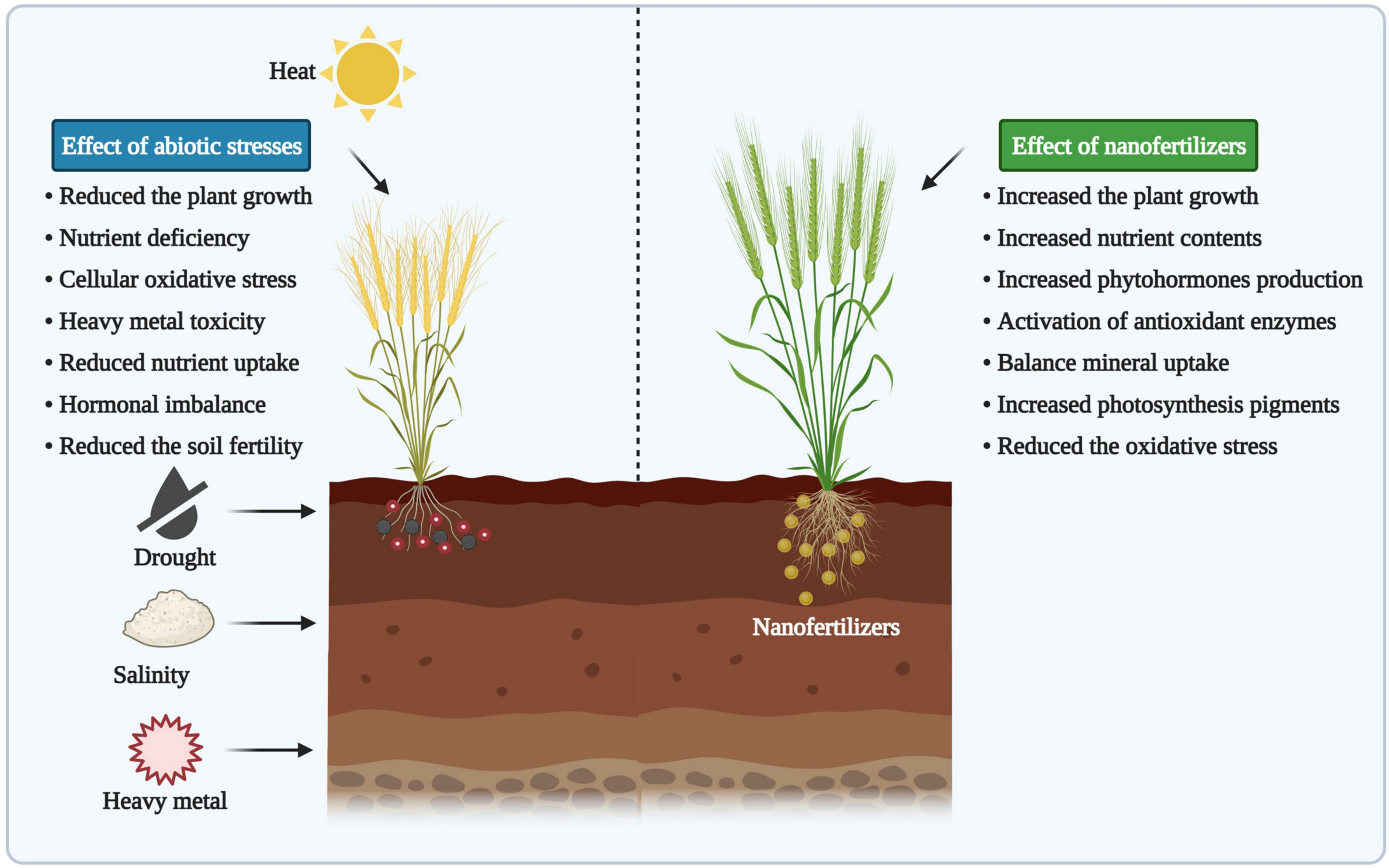
The positive effect of nanoparticles (NPs) on plant growth and development under abiotic stress conditions. The figure created using BioRender (https://biorender.com/).

Climate change has been caused by global warming, anthropogenic activities, and other unavoidable factors, and results in multiple abiotic stresses, reducing agricultural productivity and degrading natural resources (Shahzad et al., 2018). Various studies have reported a substantial reduction in the yield of many crops under abiotic stress conditions, which is mostly caused by HMs, low temperature, drought and salinity stresses (Tripathi et al., 2014; Anjum et al., 2017; Singh et al., 2017; Hussain et al., 2018b; Khan et al., 2018). Most of these abiotic stress conditions encountered by plants at various growth stages resulted in developing various defense mechanisms in plants to deal with negative physiological changes induced by environmental stresses (Ahmad et al., 2015; Jiang et al., 2016; Dimkpa et al., 2017). Several studies have revealed that abiotic stresses cause many physiological, biochemical, and molecular responses that affect several cellular processes in plants (Figure 1).

Among the abiotic stresses, soil salinity, drought and HMs contamination caused substantial reduction in crop productivity and considered as the most important threats to global crop production and food security (Godoy et al., 2021; Haider et al., 2021). Moreover, salinity and drought have been identified as damaging stressors that limit the production of many crops by inducing physiological and biochemical changes (Zia et al., 2021). These stresses restrict plant growth and productivity by imposing oxidative stress, osmotic stress, and nutritional imbalance (Shahid et al., 2020). Salinity stress causes several harmful effects on crop plants at molecular, physiological and biochemical level, which ultimately jeopardize the plant survival (Kumar et al., 2020). Accumulation of sodium (Na^+^) and chloride (Cl^−^) ions in the cytosol of the cell cause salt stress that ultimately causes considerable damage to the whole cell, resulting reduced plant growth (Rajput et al., 2019). Under drought stress, leaf stomata become closed, thus inhibiting photosynthesis in the plants, which reduces the total area of the leaf that causes a reduction in water potential and decreases plant growth by increasing osmolytes production and inducing ROS generation in plants (Ibrahim et al., 2019). The intensity and period of the drought stress are the two critical factors under drought stress, which might be correlated directly with the loss in crop productivity and economic yield (Farooq et al., 2009). However, drought combined with salinity stress causes a detrimental reduction in water potential, and decreases osmosis significantly (Hu and Schmidhalter, 2005). Abiotic stress activates agitation in plant metabolism, thus enabling reorganization of the metabolic networks to maintain important processes active (Minkina et al., 2019; Rajput et al., 2020).

HMs are considered a major class of pollutants and are very hazardous to agricultural crop plants and human health (Guo et al., 2020; Xu et al., 2021). The rapid increase in HMs contamination during recent years corresponding to the rapid industrialization, combustion of fossil fuels, atmospheric deposition, spillage of petrochemicals, mining, agricultural practices, and disposal of waste material having high metal content directly to the agricultural lands and water bodies (Noman et al., 2020a). The most common HMs present in the environmental systems are cadmium (Cd), lead (Pb), arsenic (As), nickel (Ni), chromium (Cr), cobalt (Co) and zinc (Zn). The global economic impact of HMs pollution is projected to be more than $10 billion per year (Kumar et al., 2019). Previous studies have reported the increased HMs concentrations in crops and agricultural soils as a result of significant anthropogenic and industrial waste deposition (Rizwan et al., 2019b; Noman et al., 2020b). HMs disturb redox homeostasis by stimulating the free radical formation and enhancing ROS production, which cause cellular oxidative stress by altering the cell structure, damaging membrane permeability, and proteins functionality (Sharma et al., 2012). Furthermore, HMs accumulate in the human body through the food chain and cause health problems, such as diabetes, hypertension, cardiovascular diseases, and cancer (Ahmed et al., 2021b).

## Nanotechnology for Sustainable Agriculture

**Figure 2:**
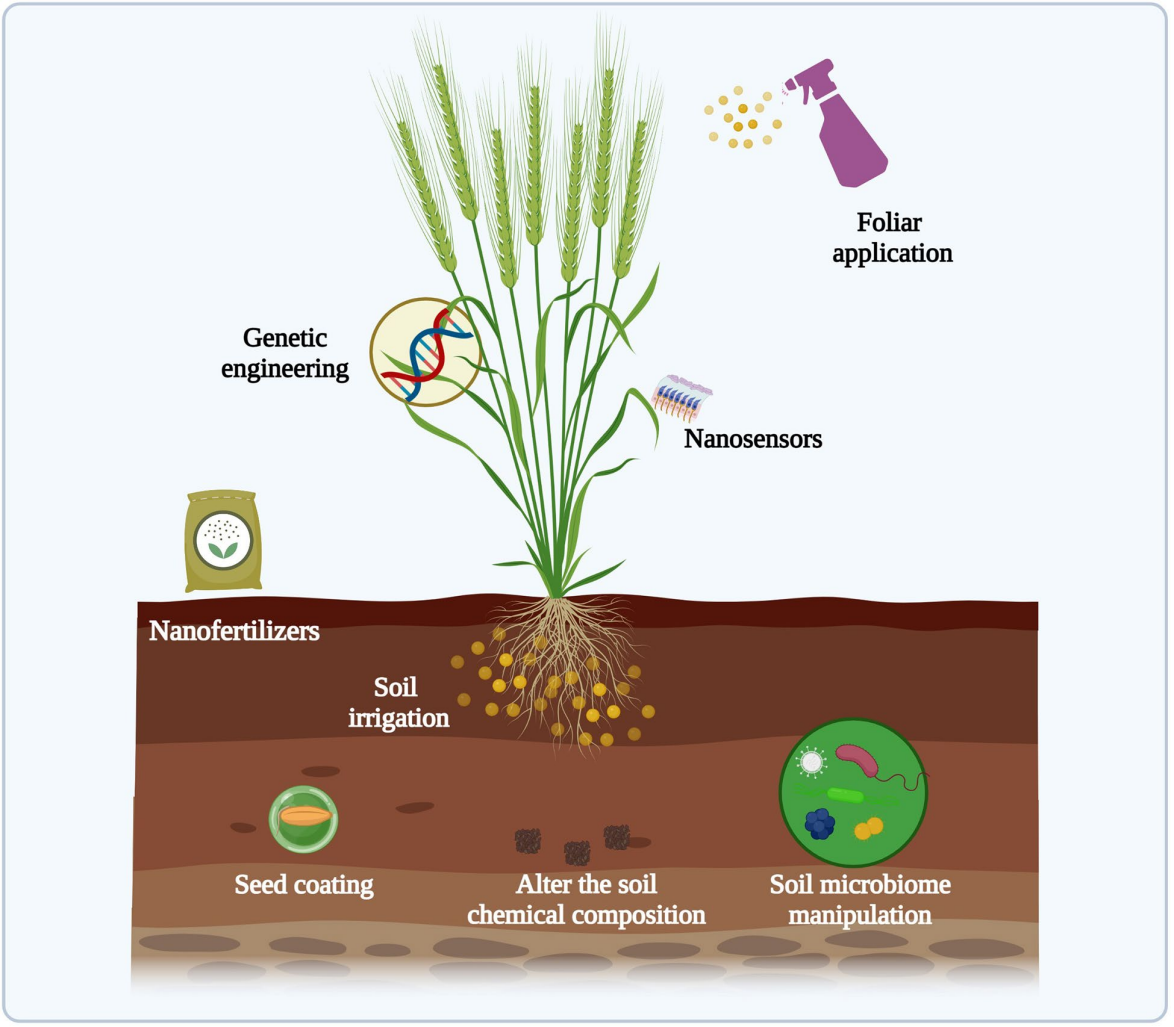
Schematic representation of potential NPs applications in plant agriculture. The figure created using BioRender (https://biorender.com/).

Nanotechnology has tremendous potential in agriculture, including mitigating climate change impacts, and improving abiotic stress management strategies (Mahakham et al., 2016). Nano-enabled technologies have been developed to promote plant growth, such as the application of nanofertilizers through different means (such as soil irrigation, foliar spray, seed coating), nano-sensors to monitor the real-time plant health condition, genetic engineering of plants to increase defense-related phytohormones and photosynthetic efficiency (Figure 2). The key benefits of using NPs compared to conventional fertilizers are the high surface-area-to-volume ratio, high contaminant removal efficiency, and efficient supply of essential nutrients for the soil health as nanofertilizers (Tripathi et al., 2015). Several studies have reported using NPs as nanofertilizers to enhance crop production under stress conditions (Iavicoli et al., 2017; Ahmed et al., 2020, 2021c).

The NPs mitigate the nutrient losses as they have more retention capacity (high surface area) for nutrients and provide potential benefits to plants (Wang et al., 2016; Ahmed et al., 2021b). The application of NPs as nanofertilizers showed efficient results in improving abiotic stress tolerance in plants by increasing the plant growth, nutrient content, phytohormones, antioxidant enzymes, and photosynthesis efficiency while reducing the cellular oxidative stress (Figure 1). Recently, iron oxide (Fe_3_O_4_) NPs have been used to enhance crop growth both under HMs contaminated soil as well as drought stress conditions (Hussain et al., 2019; Adrees et al., 2020). Furthermore, Konate et al. (2017) documented the enhanced wheat seedling growth with FeO NPs, which reduced oxidative stress induced by Cd and Pb contamination (López-Luna et al., 2015). NPs application efficiently ameliorates salt stress by decreasing salt concentration and associated toxic effects. Moreover, nano-silicon (Si) has been found to significantly ameliorate salt stress, increase seed germination, improve the antioxidant defense system, leaf turgor, and carbon-assimilation process (Haghighi and Pessarakli, 2013).

Recently, Wu et al. (2017) reported that the cerium oxide (CeO) NPs application maintained quantum yield of photosystem (PS) II and CO_2_ assimilation through ROS scavenging, particularly hydrogen peroxide, induced by abiotic stress (Horie et al., 2011). The application of titania (TiO_2_) NPs improved the activity of catalase (CAT), glutathione peroxidase (GPOX), and superoxide dismutase (SOD) and reduced oxidative stress in Duckweed (*Lemna minor*) plants (Song et al., 2012). Several beneficial and stress countering effects of NPs application in various crops have been reported, such as improved growth in *Solanum lycopersicum* L. (Shankramma et al., 2016) and *Allium cepa* L. (Anandaraj and Natarajan, 2017); *S. lycopersicum* L. (Juárez-Maldonado et al., 2016; Hernández-Hernández et al., 2018), *Oryza sativa* (Ahmed et al., 2021c), *Capsicum annuum* (Pinedo-Guerrero et al., 2017; Mozafari et al., 2018). Therefore, it can be concluded that the stress ameliorative potential of NPs can be exploited to combat various negative effects caused by abiotic stresses in crop plants.

## Uptake and Translocation of Nanoparticles in Plants

**Figure 3:**
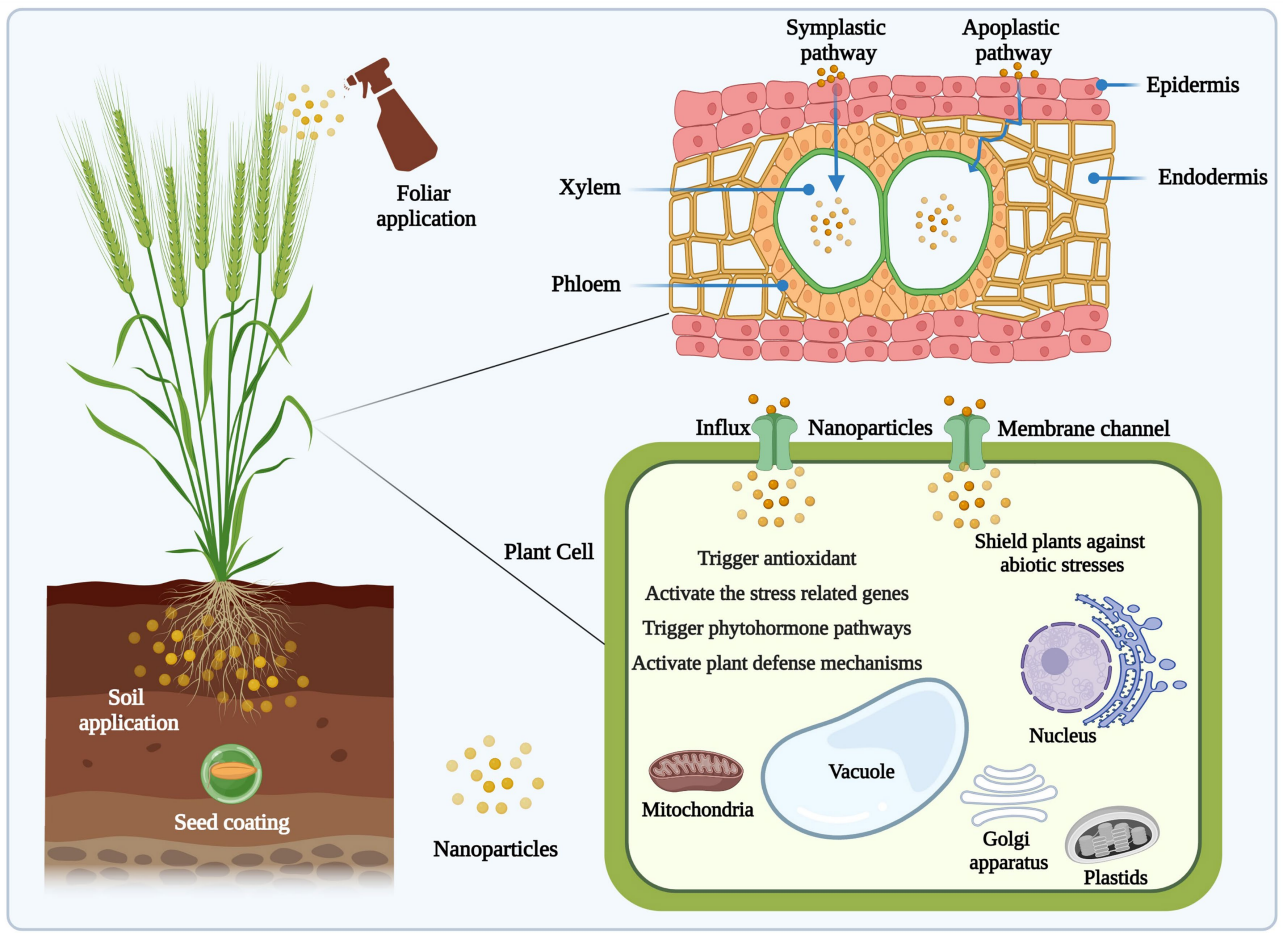
Schematic representation of NPs application approaches, uptake and translocation of NPs in plants. NPs can be delivered to plants by soil application, seed coating and foliar spray to protect plants against abiotic stresses. The figure created using BioRender (https://biorender.com/).

Different application methods, such as seed coating, soil drenching, and foliar spray are the primary means to deliver NPs into the plants (Figure 3). Plants are the main component of soil and act as a likely path for the uptake, transport, and use of NPs in the food chain (Wang et al., 2013; Dang et al., 2019). Thus, it is important to have a mechanistic insights into the uptake mechanisms of NPs in plants. In a previous study, Zhu et al. (2008) reported the uptake, translocation and accumulation of FeO NPs in pumpkin (*Cucurbita maxima*) plants after adding in the growth medium without causing any harm to the plant. Moreover, from the total balance of Fe content (67.4%), 45.4% was accumulated in roots tissues (inside and outside the root surface) and 0.6% in leaf tissue. Similarly, copper oxide NPs translocated, and distributed in maize (*Zea mays* L.) plants *via* xylem and phloem vessels (Wang, 2012). Fullerene (C70) NPs were shown to be translocated through the vascular system of rice (*O. sativa*) and can be inherited to upcoming generations (Lin et al., 2009). NPs internalized by roots/leaves and translocated *via* two pathways, i.e., apoplastic and symplastic (Huang et al., 2022). In another study, Cui et al. (2020) observed the accumulation of SiO_2_ NPs in rice cells through fluorescence and transmission electron microscopy under As-spiked condition. NPs were dispersed between cell walls and plasma membrane after overcoming the initial barrier, i.e., cell walls, and their further movement could be affected by osmotic pressure and capillary forces (Lin et al., 2009). Besides ion channels and carrier or transport proteins, such as aquaporin, NPs may enter cells by endocytosis or breaching the cell membrane barrier (Rico et al., 2011). There is no such mechanistic approach that exploits the exact location and role of NPs in plant organelles. However, further studies should be focused to explore the translocation and uptake kinetics of NPs, and extended to study the biochemical, molecular and physiological patterns of NPs uptake and transport kinetics, which is of great significance for understanding their accumulation behavior in plants.

## Role of NPs in Plants Under Abiotic Stress Conditions

Abiotic stress triggers a wide range of plant responses, varying from growth and morphological changes to crop production and yield (Kazan, 2015; Lamaoui et al., 2018). Nanotechnology has great potential to combat different abiotic stress conditions, such as HMs stress, drought stress, salt stress and heat stress, in an eco-friendly manner (Table 1).

**Table 1:** Potential application of NPs for improving abiotic stress tolerance in plants.

Nanoparticles	Plant species	Abiotic stress	Application	References
Calcium oxide NPs	Barley (*Hordeum vulgare*)	Heavy metal	Increased plant growth, photosynthesis efficiency and antioxidant enzymes	Nazir et al., 2022
Copper NPs	Wheat (*Triticum aestivum*)	Heavy metal	Increased plant growth and reduction in chromium bioavailability	Noman et al., 2020a
Iron NPs	Wheat (*Triticum aestivum*)	Heavy metal and drought stress	Improved the photosynthesis and alleviated the oxidative stress	Adrees et al., 2020
Zinc oxide NPs	Wheat (*Triticum aestivum*)	Heavy metal and drought stress	Increased the tissue dry weight and reduced the Cd accumulation	Khan et al., 2019b
Iron NPs	Wheat (*Triticum aestivum*)	Heavy metal	Improved the plant growth and reduced the oxidative stress	Rizwan et al., 2019b
Silica NPs	Wheat (*Triticum aestivum*)	Heavy metal and drought	Improved the plant growth and development	Khan et al., 2019a
Iron oxide NPs	Rice (*Oryza sativa*)	Cadmium and drought stress	Increased biomass, antioxidant enzyme contents, and photosynthesis efficiency	Ahmed et al., 2021a
Silica NPs	Cucumber (*Cucumis sativus*)	Drought and salinity	Improved the growth and productivity of cucumber plants by balancing nutrients uptake	Alsaeedi et al., 2019
Maghemite NPs	Rapeseed (*Brassica napus*)	Drought	Improved growth and reduce the drought stress	Palmqvist et al., 2017
Selenium NPs	Wheat (*Triticum aestivum*)	Drought	Enhanced the plant growth and development	Ikram et al., 2020
Iron oxide NPs	Wheat (*Triticum aestivum*)	Salinity and heavy metal	Facilitates photosynthetic pigments and restricts cadmium uptake	Manzoor et al., 2021
Maghemite NPs	*Sunflower* (*Helianthus annuus*)	Drought	Reduced drought induced by detrimental effects	Martínez-Fernández et al., 2015
Titanium oxide NPs	Tomato (*Solanum lycopersicum*)	Heat	Enhanced the plant growth and photosynthesis efficiency	Qi et al., 2013
Titanium oxide NPs	Chickpea (*Cicer arietinum*)	Cold	Increased the plant growth and antioxidant activity	Hasanpour et al., 2015
Silver NPs	Rockcress (*Arabidopsis thaliana*)	Cold	Increased expression of antioxidant activity related genes	Kohan-Baghkheirati and Geisler-Lee, 2015
Cerium oxide NPs	Soybean (*Glycine max*)	Salinity	Enhanced the plant growth by regulating photosynthesis and water use efficiency	Cao et al., 2017
Titanium oxide NPs	Broad bean (*Vicia faba*)	Salinity	Improved growth and enhance tolerance against salinity	Abdel Latef et al., 2018
Titanium oxide NPs	Moldavian dragonhead (*Dracocephalum moldavica*)	Salinity	Promote plant growth and ameliorate salinity stress	Gohari et al., 2020
Chitosan NPs	Corn (*Zea mays*)	Salinity	Mitigates the deleterious effects of salinity	Oliveira et al., 2016
Cerium oxide NPs	Cotton (*Gossypium*)	Salinity	Improved the plant growth by maintaining cytosolic K^+^/Na^+^ ratio	Liu et al., 2021
Silver NPs	Summer savory (*Satureja hortensis*)	Salinity	Improved the plant growth and germination	Nejatzadeh, 2021
Zinc oxide NPs	Safflower (*Carthamus tinctorius*)	Salinity	Enhanced the plant germination and salinity tolerance by improving the activities of antioxidant enzymes	Yasmin et al., 2021
Silicon NPs	Sweet orange (*Citrus x sinensis*)	Salinity	Improved the oxidative stress tolerance	Mahmoud et al., 2022
Gold NPs	Wheat (*Triticum aestivum*)	Salinity	Improved the plant defense systems	Wahid et al., 2022
Selenium dioxide NPs	Common bean (*Phaseolus vulgaris*)	Salinity	Enhanced the plant growth and yield	Rady et al., 2021
Cerium oxide NPs	Rice (*O. sativa*)	Salinity	Improved the crop yield by modulating the plant physiological and biochemical mechanisms	Zhou et al., 2021

## Effects of NPs in Plants Under HMs Stress

Currently, HMs contamination has been a major concern around the world (Ali et al., 2019). HMs contamination may arise due to the atmospheric deposition, mining, flourishing industrialization, waste incineration, spilling of petrochemicals, removal of high metal waste during different industrial processes (Mohammadi et al., 2018; Irshad et al., 2021a). Their long-term presence and potential toxicity in contaminated soils have been among the major environmental issues that negatively affect the sustainability of living organisms (plants, humans, and animals) (Mohammadi and Ghorbani, 2018; Rizwan et al., 2019b). Among them, Cd has greater significance in the environment due to being highly toxic to plants and humans (Khan et al., 2020; Noman et al., 2020a; Hussain et al., 2021). Accumulation of Cd in plants causes oxidative damage to the cells due to high accumulation of ROS; these negative changes in cells inhibit photosynthesis, lower ion regulation, and reduce nutrient absorption in plants, that result in reduced plant growth (Rizwan et al., 2019a; Li et al., 2020).

**Figure 4:**
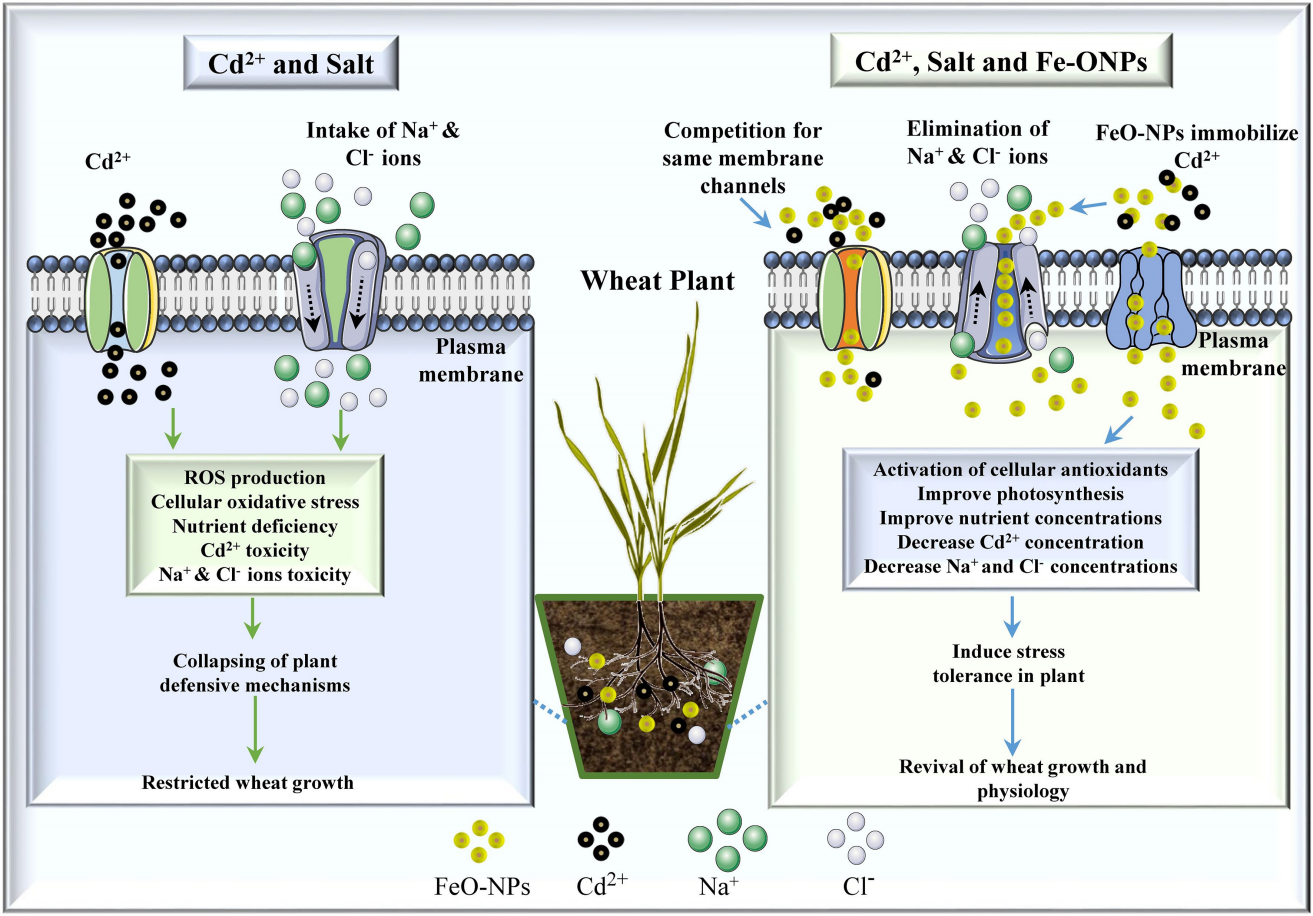
Schematic representation of iron oxide nanoparticles (FeO NPs) to alleviate the Cadmium (Cd) and salinity stress by reducing the acropetal translocation of salt and metals ions. Reproduced with permission from Manzoor et al. (2021). Copyright 2021 Elsevier. The figure created using BioRender (https://biorender.com/).

Numerous reports on the potential role of NPs in the reclamation of HMs-contaminated soil have been documented (Tripathi et al., 2015; Hussain et al., 2018a). In a recent study, Manzoor et al. (2021) demonstrated that FeO NPs ameliorated the Cd toxicity in wheat plants by improving the biomass, chlorophyll contents, and antioxidant enzymes. These NPs can decrease toxic ion accumulation in plant cells and protect from ionic stress. The effectiveness of NPs in alleviating HM toxicity in plants is due to their small size and large surface area (Figure 4). The mitigation of HM induced phytotoxicity by Si NPs has also been stated in rice (Gao et al., 2018), pea (Rehman et al., 2018), and wheat (Rehman et al., 2017). Furthermore, Konate et al. (2017) determined that FeO NPs improved the wheat plant growth by reducing the cellular oxidative stress induced by Pb and Cd toxicity. Correspondingly, magnetite NPs were also observed to increase the wheat seedlings growth by alleviating Cd and Cr toxicity (López-Luna et al., 2015). FeO NPs have recently been used effectively to enhance plant growth and to alleviate HM and drought stress simultaneously (Hussain et al., 2019; Adrees et al., 2020). However, it is necessary to develop novel nanoremediation approaches to ameliorate the negative impacts of HMs on plant growth and development.

## Effects of NPs in Plants Under Drought Stress

Drought is considered an important environmental stress that has gained the considerable interest of both environmental and agricultural scientists. It is indeed a major agricultural issue in the world, which restricts plant growth and yield. Drought stress influences various plant growth factors, negatively impacting the economic sector (Kumar and Verma, 2018). Limited moisture content reduces cell size, disturbs membrane integrity, induce oxidative stress and leaf senescence, causing a reduction in crop productivity (Tiwari et al., 2016). Previous studies have revealed that Si NPs improved the drought stress tolerance in plants. For example, drought tolerance increased in hawthorns plants supplied with Si NPs, while the defense-related physiological parameters showed variations according different drought levels and Si NPs concentrations applied (Ashkavand et al., 2018). Correspondingly, Si NPs demonstrated a good potential for post-drought plant recovery by modulating morpho-physiological properties in barley plants (Ghorbanpour et al., 2020). Alsaeedi et al. (2019) reported that Si NPs enhanced cucumber growth and yield under water-deficient and saline conditions.

Chitosan NPs have increased relative water content, photosynthetic rate, CAT, SOD activities, yield, and biomass of wheat plants under drought stress (Behboudi et al., 2019). Foliar application of Fe NPs was reported to alleviate drought stress effects on safflower cultivars (Davar Zareii et al., 2014), while soil application of CeO NPs significantly improved plant growth at 100 mg/kg and increased the photosynthetic rate by regulating the water use efficiency in soybean (*Glycine max*) plants (Cao et al., 2017). The negative effects of drought stress on lentils (*Lens culinaris* Medic.) plants was reduced by the application of silver NPs (Das and Das, 2019). Sun et al. (2017) reported Si NPs-assisted delivery of abscisic acid as an effective management strategy to improve drought resistance in *Arabidopsis thaliana*.

## Effects of NPs in Plants Under Salt Stress

Soil salinity substantially reduce crop production worldwide by inducing ionic toxicity and disturbs ionic homeostasis (Parihar et al., 2015; Etesami et al., 2021). Saline conditions lead to the deposition of Na^+^ and Cl^−^ ions in plant cells, which cause ionic imbalance and toxicity (Shabala and Cuin, 2008; Khan et al., 2021). In plants, salt stress immensely induces K^+^ efflux from leaf mesophyll cells and enhances the deposition of Na^+^ in cytosols. Hence, CO_2_ assimilation is reduced in saline environments, thus reducing growth rate and production (Parida and Das, 2005). The application of different NPs is an alternative approach to combat the salt stress, which mitigates the accompanying toxicity impacts. In a previous study, Si NPs significantly improved the seed germination, carbon assimilation, leaf turgor, and antioxidant defense system in cherry tomatoes (*S. lycopersicum* L.) plants under salt stress conditions (Haghighi and Pessarakli, 2013).

Similarly, Manzoor et al. (2021) reported that FeO NPs alleviated the salt stress by improving the growth, chlorophyll contents, and antioxidant enzymes in wheat plants. These NPs can decrease salt ions accumulation in cells and protect plants from ionic stress (Figure 4). Under salt stress, improvement in seed germination and seedlings growth was reported in Si NPs-supplied lentils (*L. culinaris* Medic.) plants (Sabaghnia and Janmohammadi, 2015). In another study, Ye et al. (2020) revealed that seed priming of manganese NPs control salinity stress by modulating molecular responses in Pepper (*C. annuum* L.) plants. Zhao et al. (2019) observed that the multi-walled carbon nanotube amendments improved the salinity tolerance in rapeseed (*Brassica napus* L.) by decreasing the ROS production, thiobarbituric acid and Na^+^/K^+^ ratio. However, more research is required at physiochemical and molecular levels to explore the mode of actions of NPs to improve the salinity tolerance in plants.

## Effects of NPs in Plants Under Heat Stress

The constantly rising temperature is considered as one of the most damaging stress among the ever-changing environmental factors (Ohama et al., 2017). Heat stress enhances the ROS production and induces oxidative stress, resulting in the membrane lipids degeneration, disturb cellular homeostasis and impairment of different metabolic processes, which finally cause cells death in crop plant (Savicka and Škute, 2010). Moreover, heat stress photosystem II disruption, electron flow disruption, carbon fixation diminution and induces chlorophyll degradation, which disrupt photosynthesis process and decreased the plant growth (Li et al., 2021). Recent advancements in nanotechnology have modernized agriculture system with promising application to improve the plant growth and development under stress condition (Rana et al., 2021). Several studies revealed the potential application of NPs to improve the heat stress tolerance in crop plants (Wu and Wang, 2020; Thakur et al., 2021).

In a previous study, Haghighi et al. (2014) observed the application of selenium (Se) NPs significantly reduced the heat stress by improving the chlorophyll content, hydration potential, and growth of tomato plants. Similarly, TiO_2_ NPs amendments significantly reduced the heat stress by stomatal opening in tomato plants (Qi et al., 2013). El-Saadony et al. (2021) reported that the application of biologically synthesized Se NPs (100 μg/ml) improved the wheat growth by increasing heat stress tolerance in plants. In another study, Iqbal et al. (2019) also revealed that silver NPs significantly increased the morphological attributes of wheat plants under heat stress condition. Overall, the application of metallic NPs as nanofertilizers can be used to improve the plant tolerance to heat stress for the sustainable agriculture.

## Conclusions and Future Perspectives

In the last few years, a major concern of the research community is to overcome the negative effects of abiotic stresses on crop production. This review has revealed the potential of NPs to protect crop plants from different abiotic stresses and the mechanisms of NPs accumulation in plants. The application of NPs significantly improved the abiotic stress tolerance in plants by improving the cellular antioxidants, nutrient uptake, photosynthesis efficiency, and regulation of biochemical/molecular mechanisms. Although nanofertilizers can provide a cost-efficient approach for improving the abiotic stress tolerance in plants by providing essential nutrients, however, their extensive use has prompted potential concerns about their negative effects on the ecosystem. In the future, more research insights are required to explore the interactions between NPs and plants to study the adverse effects of residual NPs on the environment systems. The transition from the laboratory to the field is incredibly difficult without promising results from field trials. Further studies considered necessary before starting the field application, such as long-term effectiveness of NPs in field conditions, ecotoxicological risk factors, and the impact of NPs on the metabolome, proteome, metagenome, and transcriptome of the plant and soil systems. The industrial sectors should be established to scale up nanoproducts, train farmers on nanoformulation application, develop application procedures and manage the regulatory landscape. Moreover, future research should emphasize on the designing of cost efficient, nontoxic, self-degradable, and eco-friendly NPs by using green approaches. We anticipate that this review will be useful to develop effective nano-enabled techniques in agriculture sectors to manage the global problem of food security caused by different abiotic stresses.

## Author Contributions

NM and LA: conceptualization, visualization, and writing—original draft. TA: writing—review, validation, and editing. MN, MA, and MS: conceptualization, writing—review, and editing. SO and KR: writing—review and editing. GW and HZ: conceptualization, funding acquisition, supervision, writing—review, and editing. All authors contributed to the article and approved the submitted version.

## Funding

This work is financially supported by the 2115 Talent Development Program of China Agricultural University (00109012) and the Scholarship of the “National Thousand (Young) Talents Program” of China (D1201040).

## Conflict of Interest

The authors declare that the research was conducted in the absence of any commercial or financial relationships that could be construed as a potential conflict of interest.

## Publisher’s Note

All claims expressed in this article are solely those of the authors and do not necessarily represent those of their affiliated organizations, or those of the publisher, the editors and the reviewers. Any product that may be evaluated in this article, or claim that may be made by its manufacturer, is not guaranteed or endorsed by the publisher.
